# Disease Mechanisms of Multiple System Atrophy: What a Parallel Between the Form of Pasta and the Alpha-Synuclein Assemblies Involved in MSA and PD Tells Us

**DOI:** 10.1007/s12311-022-01417-0

**Published:** 2022-06-03

**Authors:** Ronald Melki

**Affiliations:** Institut Francois Jacob (MIRCen), CEA and Laboratory of Neurodegenerative Diseases, CNRS, 18 Route du Panorama, 92265 Fontenay-Aux-Roses, France

**Keywords:** Protein folding, Alpha-synuclein aggregation, Fibrillar polymorphism, Multiple system atrophy, Parkinson’s disease

## Abstract

Intracellular deposits rich in aggregated alpha-synuclein that appear within the central nervous system are intimately associated to Parkinson’s disease and multiple system atrophy. While it is understandable that the aggregation of proteins, which share no primary structure identity, such as alpha-synuclein and tau protein, leads to different diseases, that of a given protein yielding distinct pathologies is counterintuitive. This short review relates molecular and mechanistic processes to the observed pathological diversity associated to alpha-synuclein aggregation.

## Introduction

The aggregation of the proteins Tau, A-beta, alpha-synuclein, PrP, Huntingtin, and serpins, into assemblies of fibrillar nature is intimately associated to tauopathies, such as Alzheimer’s disease; synucleinopathies, such as Parkinson’s disease; spongiform encephalopathies, such as Creutzfeldt-Jacob disease; Huntington’s disease; and different forms of dementias. These proteins share no primary structure identity, and this may account for the different diseases they cause or are associated to given that it is widely accepted that unrelated protein aggregates compromise cellular proteostasis and integrity in different manners [1]. Alpha-synuclein aggregation is involved in Parkinson’s disease, Lewy body dementia, and the two forms of multiple system atrophy. Alpha-synuclein is not the only protein to bear this property, Tau protein aggregation is involved in Alzheimer’s disease but also Pick’s disease, progressive supranuclear palsy, globular glial tauopathy, aging-related tau astrogliopathy, chronic traumatic encephalopathy, argyrophilic grain disease, and primary age-related tauopathy. How the aggregation of one given protein leads to different diseases is a priori puzzling. There must be explanations for this fact. It could be that alpha-synuclein aggregation occurs only in a set of neuronal cell populations that are permissive to aggregation, which suffer and degenerate afterward leading to specific phenotypes. It could also be that the aggregates alpha-synuclein forms in all neuronal cells or in different cell populations are different, thus causing different diseases. It could further be that alpha-synuclein aggregates forming in defined neuronal cells target and affect specific neuronal cell populations via their prion-like properties. It is worth keeping in mind that none of these possibilities are exclusive. This short review provides plausible molecular and mechanistic events that may account for the observed pathological diversity.

## Structural diversity of alpha-synuclein amyloids

Alpha-synuclein is a relatively small “natively unfolded” protein. The term natively unfolded reflects alpha-synuclein molecule intrinsic dynamics. Indeed, this protein in total or part adopts multiple conformation, not necessarily extended, which does not allow determining an average conformation unless upon binding a ligand [2–13]. The ensemble of tertiary structures or conformational states alpha-synuclein populates is immense. Indeed, if we assume that each amino acid residue within alpha-synuclein can adopt a limited number of conformations, for example 3 (1 trans and 2 gauche) around the 139 peptide bonds within the protein, with only 2 torsions each, the number of possible conformations that alpha-synuclein could adopt would surpass 3^278^ (3^139×2^) conformations. The different conformations within the ensemble of tertiary structures are in equilibrium, meaning that each conformation is populated for a given time [14]. If each conformation is populated for a very small fraction of a second, e.g. 10^−12^ s, it would take a single molecule over the age of the universe to populate this limited set of possible conformations. The concentration and lifespan of each conformation are specific to the tertiary conformation and are defined by intramolecular interactions between amino acid residues stabilized by hydrogen bonds and electrostatic and hydrophobic interactions. The latter depend on the amino acid composition of the protein, the distribution of the amino acid residues within the protein primary structure, and the chemical and physical conditions surrounding the protein. This is why the ensemble of conformations alpha-synuclein can explore is several orders of magnitude smaller than the number indicated above. It is worth adding that mutations within alpha-synuclein, such as the ones reported in familial early onset parkinsonism, affect the intramolecular interactions between amino acid residues within alpha-synuclein and because of that the ensemble of conformations mutant alpha-synuclein molecules explore.

Because of its dynamics, the probability for wild type or mutant alpha-synuclein to populate conformers exposing amino acid stretches that allow them to establish well-defined inter-molecular interactions with molecules that are in a compatible conformation is far from negligible at any time. This allows the monomeric protein to form oligomeric species. As upon the crystallization of a protein, the nature of the interactions allowing the formation of quaternary structures such as dimers, trimers, and higher molecular weight oligomeric species between molecules defines the stability of the assemblies this protein forms [15]. When a molecule adopts conformations incapable of establishing stable and highly complementary interaction with the oligomer elongating tips, it cannot add on and be subsequently incorporated within the seed. However, a given protein in different conformations can establish different complementary interactions with molecules in compatible conformation. Thus, assemblies, including crystals, made of the same protein in different conformations can form. What is true for a generic protein applies to alpha-synuclein.

Similar to most of the proteins whose aggregation is associated to neurodegenerative diseases, alpha-synuclein adopts beta-strand secondary structure–rich conformations. While the distribution of these beta strands is highly dependent on wild-type or mutant alpha-synuclein primary structure, they form transiently because of the protein dynamics. The establishment of numerous hydrogen bonds between alpha-synuclein molecules allows their highly ordered stacking into assemblies of fibrillar shapes named amyloids. The term amyloid signifies starch like. This term was used in the nineteenth century to refer to deposits within the brain that stain pale blue with iodine and violet upon treatment with sulfuric acid as do starch deposits in plants. Amyloid fibrils are defined as fibrillar polypeptide aggregates with cross-beta conformation, a structure where the hydrogen bonds between two consecutive sheets are oriented parallel to the main fibril axis while the constituting beta-strands are oriented transversely to the main fibril axis [16, 17]. This type of structure gives rise to a characteristic pattern of reflections in X-ray diffraction experiments consisting of a conserved 4.6–4.8 Angström meridional spacing and an equatorial spacing of about 10 Angström. The 4.6–4.8 Angström reflection comes from the distance between two hydrogen bonded strands and is invariant as it depends on the geometry of the polypeptide backbone. It is referred to as the “main chain spacing.” The equatorial reflection at about 10 Angström comes from the packing distance between two juxtaposed beta-sheets [18, 19]. This distance can vary with the polypeptide amino acid composition as it depends on the orthogonal protrusion of the amino acid side chains from the plane of the sheet. It is worth noting that this reflection is not observed when the inter-sheet spacing is not regular.

As the organization of polypeptide stretches into beta strands within wild-type and mutant alpha-synuclein molecules is transient because of the protein dynamics, different beta-strand–rich conformations form and disappear. Among these, different conformers are compatible with the formation of structurally distinct amyloid fibrils. All resulting fibrils have the characteristic 4.6–4.8 and ~ 10 Angström reflections. The fibrils may also exhibit additional reflections originating from highly ordered domains stacks, when they exist.

**Fig. 1:**
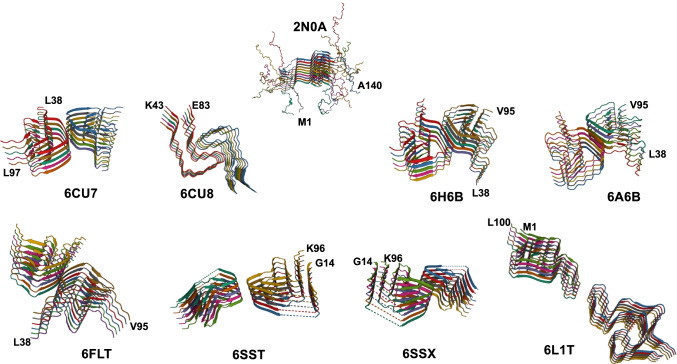
Structure of the amyloid core of the different fibrillar polymorphs alpha-synuclein forms under different experimental conditions and post-translational modifications. The structure of fibrillar alpha-synuclein obtained by solid-state NMR (pdb ID# 2n0a [30] is represented on the top. The structures of full-length or truncated wild type or mutant alpha-synuclein derived from cryo-electron microscopy are represented on the two bottom lines (PDB id# 6cu7 [24, 25], 6h6b [22], 6flt [22], 6a6b [24, 25], 6cu8 [24, 25], 6sst, 6ssx [23], 6l1t [31]. The respective pdb identities are given. The identity of the amino acid residues located at the N- and C-terminal sides of alpha-synuclein molecule within the resolved fibrillar structures are indicated

**Fig. 2:**
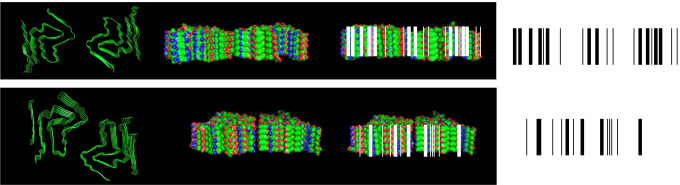
The surfaces of different fibrillar polymorphs that result from the highly ordered pile up of alpha-synuclein molecules within fibrillar assemblies define a molecular bar code. The structures used to illustrate the different surfaces are 6sst, 6ssx [23] viewed from top (left) and side (middle left). Basic residues are in blue, acidic residues in red, and uncharged residues in green. In the bar codes, basic and acidic residue piles are represented by thick and thin lines, respectively (two panels on the right)

As indicated above, the tertiary structures wild-type or mutant alpha-synuclein adopts are the consequences of intramolecular interactions between amino acid residues stabilized by hydrogen bonds and electrostatic and hydrophobic interactions. Those intramolecular interactions are highly dependent on the chemical and physical conditions surrounding the protein (pH, viscosity, ionic strength, nature of ions etc.…). Changing the conditions under which alpha-synuclein forms amyloid fibrils allows populating different alpha-synuclein tertiary structures or fold ensembles. The different tertiary structures or folds any given amyloid forming protein can adopt define the way the molecules stack into fibrils, the intermolecular interfaces between any two consecutive protein molecules and the number of intermolecular H-bonds within these interfaces. This affects mostly fibril growth through the incorporation of molecules in compatible conformations. Different folds for a given protein define the surfaces of the stacks they form. The latter allow or not defined interactions between different stacks of the protein in the same or different conformations yielding fibrils made of multiple protofilaments. This further governs fibril growth rates, nanomechanical properties, processing, and degradation by the cellular machinery in charge of their clearance. Thus, the intrinsic architectures of fibrils made from one given protein, e.g., alpha-synuclein (Fig. 1), are defined by the amyloid forming folds its constituting protein populates [20], [21–28]. The involvement of different amino acid stretches in the amyloid core of alpha-synuclein fibrils exhibiting distinct intrinsic architectures implies they possess unlike surfaces. Indeed, the amyloid fold and scaffold define the amino acid stretches composing the solvent-exposed polypeptide chains and their distribution in space at the external surfaces of the fibrils, the surfaces through which they interact with ligands ranging from small molecules to cell components [29]. When schematized, these surfaces resemble molecular bar codes (Fig. 2).

## Alpha-synuclein Amyloid Structural Diversity and Differential Tropism and Seeding

The amino acid residues and peptide chains that are exposed at the growing tips of the fibrils define the rate at which they elongate by incorporation of monomeric alpha-synuclein molecules in compatible conformation(s). Those exposed to the solvent on the sides of alpha-synuclein fibrils and their spatial distribution determine what membranous components, in particular at the plasma membrane, they can interact with. The presence, identity, and density of those receptors at the surface of neuronal cells define whether one amyloid fibrillar form of alpha-synuclein can bind to a given cell and the tropism of the fibrils toward those cells. These receptors are the extracellular matrix, phospholipids, and membrane proteins. The amino acid residues and peptide chains that are exposed on the sides of structurally distinct fibrils also define whether alpha-synuclein may be post-translationally modified and, as a consequence, their interactomes that depend on post-translational modifications. Finally, the amino acid residues and peptide chains that are exposed on the sides of alpha-synuclein amyloids determine with what efficiency they are degraded by the cellular clearance machinery and/or extracellular proteases while those exposed at their growing ends define the rate at which incorporated alpha-synuclein molecule is lost from their tips.

Alpha-synuclein fibrils binding to the cell membrane have been shown to affect membrane fluidity and geometry/curvature [32]. The lateral diffusion of alpha-synuclein fibrils in the plane of the plasma membrane together or independently of membranous components such as receptors, channels, and adhesion molecules, contributes to the build-up of large fibrillar clusters and the redistribution and/or coalescence of protein partners at the surface of the cell [30–32, 35]. The plasma membrane interactome of a single fibrillar alpha-synuclein polymorph has been extensively assessed allowing the identification of receptors at neuronal cells surfaces such as α3 subunit of neuronal sodium/potassium pump, agrin, apolipoprotein E, catenin delta-2, glypicans, liprin, neurexin subunits, Na^33^–^36^^+^/ K^+^-transporting ATPase, amyloid β precursor-like protein 1, voltage-dependent anion-selective channel protein, and the glucose related protein of 78 kDa [34, 37–39]. Assessment of the interaction of structurally distinct alpha-synuclein fibrillar polymorphs with neuron membranes revealed differential binding and redistribution of the synaptic α3 subunit of neuronal sodium/potassium pump, NMDA, and AMPA receptors, thus demonstrating the importance of amino acid residues and peptide chains constituting structurally distinct alpha-synuclein fibril sides [35].

Structurally distinct alpha-synuclein fibril differential binding to neurons appears related to differential seeding. The fibrillar polymorph that bound best seeded to the highest extent [35]. Seeding of endogenous alpha-synuclein is dependent on (1) fibril intrinsic seeding propensity; (2) fibril binding to the plasma membrane; (3) uptake of the fibrils, mostly by endocytosis; and (4) fibril escape from the endo-lysosomal compartment and/or resistance to degradation. The distinct fibrillar alpha-synuclein strains were fragmented to an average length of 40–50 nm (compatible with endocytosis) in Shrivastava et al. [35] so that differential uptake does not account for the different seeding propensities. Thus, differential seeding reflects either differential (1) binding that has been demonstrated as indicated above, (2) resistance to the cellular clearance machinery and/or escape from the endo-lysosomal compartment, or (3) growth rates of structurally distinct alpha-synuclein fibrils via recruitment of endogenous monomeric alpha-synuclein at rates highly dependent on the abundance of the conformation that can establish highly complementary interactions with their ends within neurons. All of the processes listed above depend on the structure and surfaces of distinct alpha-synuclein amyloids.

Besides the increased seeding reported in neurons overexpressing alpha-synuclein after *SNCA* gene duplication/triplication or mutations in *SNCA*, differential seeding appears to depend on *SNCA* gene expression level in neuronal cell populations. Hippocampal and dopaminergic neurons express for instance alpha-synuclein to the highest extent while the expression levels are low in striatal neurons and oligodendrocytes [40–42].

## Alpha-Synuclein Amyloid Structural Diversity at the Synapses and Within Neuronal Cell Cytosol

Synapses are dynamic structures that constantly remodel in an activity and signaling manner [43]. An alpha-synuclein fibrillar amyloid-dependent redistribution of synaptic membrane proteins was reported [35] suggesting differential signaling between neurons and neuronal network dysfunction [44].

After take up by neuronal cells, structurally distinct alpha-synuclein amyloids interact with cytosolic components ranging from organelles to proteins whose nature, as for membranous proteins, is dictated by fibril surfaces. Thus, different alpha-synuclein fibrillar polymorphs redistribute and or trap into a non-functional state different partner proteins. They also compromise to different extents the integrity of intracellular membranous compartments [44–49]. In addition, alpha-synuclein amyloid structure and the probability with which the tertiary structures compatible with fibril ends are populated define the rate at which functional monomeric alpha-synuclein is exhausted in neurons upon elongation and multiplication of the fibrils and contribute to the speed at which pathology progresses with time.

## Relationship Between Alpha-Synuclein Amyloid Fibril Diversity and the Nature of Pathology

Intracerebral or systemic injections of structurally distinct alpha-synuclein amyloids into experimental animals gave rise to different neuropathology with features that resemble Parkinson’s disease (PD) and multiple system atrophy (MSA) [50]. Structurally distinct alpha-synuclein fibrils seeded the aggregation of their counterparts to different extents and spread differentially within the central nervous system yielding distinct propagation patterns [51]. Additional evidences supporting a relationship between alpha-synuclein amyloid fibril diversity and the nature of pathology come from methods where pathogenic alpha-synuclein-rich aggregates from the central or peripheral nervous system of patients suffering from different synucleinopathies were used to seed the aggregation of monomeric alpha-synuclein in test tubes. No seeding was observed under this experimental setup when tissue homogenates from control patients were used. Seeded aggregation occurred when equivalent tissues from PD, MSA, and DLB patients were used. The resulting, templated, alpha-synuclein fibrils exhibited distinct disease-specific structural features [52–55]. Thus, as described in this short review, the structures alpha-synuclein molecules adopt within the amyloid fibrils this protein form dictate the scaffold, e.g., the shape of the fibrils as well as the amino acid stretches exposed on their surfaces. The latter define their interactomes. The full interactomes of structurally distinct alpha-synuclein pathogenic amyloids whether in the cytoplasm or at the plasma membrane are far from being known. A number of extracellularly exposed membranous components have been identified. The presence and abundance of these proteins on the surface of neuronal cells define the tropism of distinct alpha-synuclein amyloids, propagating in a prion-like manner, for different neuronal cell populations within the central nervous system. In other words, their target cell populations. Further identification of the plasma membrane interactomes of fibrillar alpha-synuclein polymorphs will bring additional insights into their tropism for different neuronal cell populations.

## Alpha-Synuclein-Rich Inclusions in Oligodendrocytes in MSA

Alpha-synuclein-rich deposits in oligodendrocytes are observed in the brain of patients suffering from MSA besides the pathological inclusions within neurons that characterize all synucleinopathies. The origin of these oligodendroglial inclusions (GCIs) is as yet unknown. They have been hypothesized to result from the uptake of aggregated alpha-synuclein, released from neurons, by oligodendrocytes, or by overexpression of *SNCA* mRNA in pathological condition specific to MSA [56, 57]. For long, mature oligodendrocytes have been considered to lack alpha-synuclein [58–60]. Solid evidence for the presence of *SNCA* mRNA in oligodendrocytes has been brought suggesting that alpha-synuclein may be expressed in these cells [40]. It is nonetheless unclear whether alpha-synuclein expression level within oligodendrocytes exceeds the critical concentration for de novo aggregation of the protein. The exogenous origin of GCI is thus to be considered. Oligodendrocytes have been shown to bind and take up exogenous fibrils [44, 61]. Different neuronal cells have been shown to process exogenous fibrils to different extents [62], and different strains have been shown to bind neuronal cells and seed endogenous alpha-synuclein to different levels. Thus, oligodendrocytes may take up and accumulate aggregated alpha-synuclein because of their inability to degrade the aggregates. As indicated above, this is expected to have deleterious consequences for oligodendrocytes because of the redistribution of plasma membrane proteins at the surface of those cells and the sequestering of oligodendroglial cytosolic proteins. This is not the only plausible scenario. Indeed, endogenous alpha-synuclein seeding has been correlated to upregulation of SNCA expression in neurons [63]. This is where the discovery of *SNCA* mRNA in oligodendrocytes and the finding that alpha-synuclein is able to form different strains become crucial. Indeed, it is not unreasonable to envisage differential processing of distinct alpha-synuclein strains in oligodendrocytes. It is further reasonable to conceive exogenous strain-mediated seeding of endogenous oligodendroglial alpha-synuclein despite the low levels of the proteins within those cells. Such a scenario would lead to the persistence and/or growth of GCIs. Alternatively, as the intracellular conditions within oligodendrocytes are different from those in other neuronal cells, take up of pathogenic alpha-synuclein aggregates by oligodendrocytes and the yet to be demonstrated upregulation of *SNCA* expression within these cells may yield novel strains [64].

## Conclusion

The robustness of the evidences for the existence of alpha-synuclein aggregates with distinct structures and/or characteristics in different synucleinopathies is increasing [26, 52–55, 63, 65, 66]. Direct or indirect interactors of aggregated alpha-synuclein within those pathogenic deposits are also being identified [63, 67–76]. These studies and the very complex results they yield are crucial for understanding the onset and differential progression of distinct synucleinopathies.

For simplicity, this review was focused on findings supporting the structure-pathology relationship that was derived from experiments performed mostly in vitro with pure structurally distinct alpha-synuclein fibrillar assemblies. The artificial approaches this short review focused on show that the mechanism of alpha-synuclein aggregation into distinct fibrillar assemblies and the molecular processes driven by the differential interactions of the resulting fibrils are at the origin of a sequential deleterious scenario. Indeed, it has been established that the binding of structurally distinct alpha-synuclein amyloids to neuronal cells plasma leads to differential redistribution of essential membrane proteins, synaptic remodeling, and impaired neuronal activity. Structurally different alpha-synuclein fibrils further trigger noxious changes with the differential seeded aggregation of endogenous alpha-synuclein that leads to differential loss of function of cytosolic proteins after their trapping within the pathogenic aggregates. The latter also compromises mitochondrial function [44, 77–79].

**Fig. 3:**
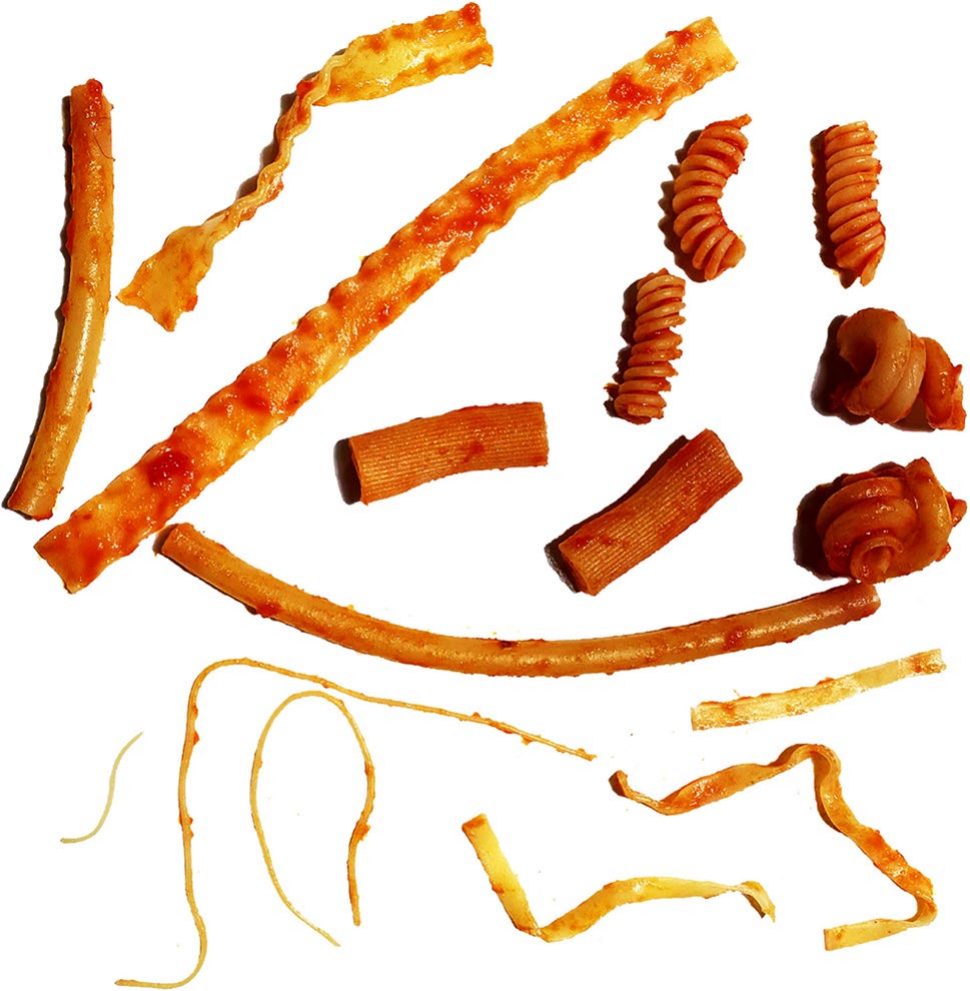
Parallel between the form of pasta and the alpha-synuclein assemblies involved in MSA and PD. Pastas exhibit different shapes as fibrillar alpha-synuclein polymorphs do. Some are thick, some are thin, some are twisted, and some are flat. The different shapes pastas exhibit define how they interact with their environment, here, the tomato sauce. Some interact very efficiently with the sauce with high binding; they look dark, others much less efficiently and look pale

Pastas illustrate in realistic manner the molecular processes described in this review (Fig. 3). Pastas are made of the same component, most often cereals and predominantly durum wheat. The pasta named spaghetti differs in shape from linguine, vermicelli, fusilli, penne, rigatoni, macheroni, etc.… The different shapes of pastas define how they interact with the environment they face, e.g., the sauce they are into. Some interact very efficiently with the sauce with high binding, others much less efficiently. The properties of different sauces also play a critical role in their differential interaction with pastas exhibiting different shapes.
